# Relationship Between Vitamin D Levels and β Cell Function and Insulin Resistance

**DOI:** 10.7759/cureus.33970

**Published:** 2023-01-19

**Authors:** Cem Ozcan, Demet Corapcıoglu, Ethem Turgay Cerit

**Affiliations:** 1 Internal Medicine, Yüksek İhtisas University School of Medicine, Ankara, TUR; 2 Endocrinology and Metabolism, Ankara University School of Medicine, Ankara, TUR; 3 Endocrinology, Ankara Memorial Hospital, Ankara, TUR

**Keywords:** 1.25(oh)2d3, insulin sensitivity, insulin resistance, prediabetes, diabetes, 25(oh)d, vitamin d

## Abstract

Background

This study aimed to determine the relationship between vitamin D levels and β cell function and insulin resistance in patients with diabetes, glucose tolerance disorder, or impaired fasting glucose.

Methodology

A total of 75 outpatients (55 females and 20 males) between the ages of 30 and 65 years were included in the study. There were 25 healthy individuals, 25 individuals with prediabetes, and 25 individuals with diabetes. The Homeostasis Model Assessment (HOMA) score was used to evaluate insulin resistance.

Results

The mean levels of vitamin 25(OH)D among the groups included in the study were 35 ± 16.9 nmol/L in the control group, 44.5 ± 34.5 nmol/L in the prediabetes group, and 35.7 ± 13.1 nmol/L in the diabetes group. There were no significant differences. The mean level of vitamin 1.25(OH)_2_D_3 _was 15.95 ± 8 pg/mL in the control group, 18.4 ± 7.5 pg/mL in the prediabetes group, and 21.5 ± 7.9 pg/mL in the diabetes group. While the levels of vitamin 25(OH)D were similar between the groups, the levels of vitamin 1,25(OH)_2_D_3 _ were significantly higher in the diabetes group. Considering all individuals, no significant difference was found between the vitamin 25(OH)D and glucose levels at minutes 0, 30, 60, 90, and 120. While there was a significant positive relationship between the 1,25(OH)_2_D_3_ vitamin and glucose levels at minutes 0, 30, 60, and 90, there was no significant relationship between the levels at minute 120. When the 1,25(OH)_2_D_3_ vitamin and HOMA insulin resistance and HOMA β scores were compared, a significant positive relationship was found between the 1,25(OH)_2_D_3_ vitamin and HOMA β levels.

Conclusions

In our study, there was no significant difference between the groups (control, prediabetes, and diabetes) in 25(OH)D levels. Similarly, there was no significant relationship between the 25(OH)D levels and insulin sensitivity and resistance between the groups. The positive relationship identified between the 1,25(OH)_2_D_3_ vitamin levels and the glucose concentration at minutes 0, 30, 60, and 90 and the higher 1,25(OH)_2_D_3_ vitamin levels in the diabetes group compared to the control group in our study can be interpreted as the effort of the organism to prevent glucose-induced β-cell apoptosis.

## Introduction

Vitamin D, unlike traditional vitamins, is synthesized in the body and thus classified as a hormone. Today, vitamin D deficiency is known to play a role in the formation of autoimmune diseases, inflammatory bowel disease, rheumatoid arthritis, multiple sclerosis, diabetes, many types of cancer, and heart diseases [1-4]. A relationship between type 2 diabetes mellitus (DM) and impaired glucose tolerance (IGT) and vitamin D deficiency was reported years ago [5]. 

Many studies have addressed the role of vitamin D on the endocrine pancreas and particularly on beta (β) cells [6]. In addition to 1,25(OH)_2_D_3_ receptors, other pathways have been identified in β cells. It has been determined that 1,25(OH)_2_D_3_ affects β cells due to the presence of vitamin D-calcium-dependent protein (Calbindin D). Calbindin D expression has been shown to protect β cells from cytokine-dependent death [7,8].

The 25(OH)D concentration was found to be lower in individuals with type 2 DM compared to the control groups without type 2 DM [8]. In patients with type 2 DM who do not have additional risk factors, low 25(OH)D levels pose a risk for diabetes. Therefore, 1,25(OH)_2_D_3_ is thought to play an important role in glucose metabolism by both increasing insulin secretion and preventing cytokine-dependent death of β cells [9].

The purpose of this study is to determine the relationship between vitamin D levels, β-cell function, and insulin resistance in patients with diabetes, glucose tolerance disorder, or impaired fasting glucose.

## Materials and methods

Patient demographics

The study included 75 outpatient cases (55 females and 20 males) in the 30-65-year age range, 25 with normal, 25 with prediabetes (IAG and IGT), and 25 with diabetes. The cases were initially divided into four groups according to the results of the oral glucose tolerance test (OGTT) and fasting blood glucose levels. Among these cases, 25 cases with impaired fasting glucose (IFG) and IGT were categorized as prediabetes, 25 cases as diabetes, and 25 cases as control. Individuals in the 30-65-year age range, those not on oral antidiabetic or insulin treatment, those not using medications that can affect Ca^2+^ metabolism (Ca and vitamin D, bisphosphonates, calcitonin, selective estrogen receptor modulators, antiepileptics, thyroid hormone drugs, and steroids), and those without insulin resistance and additional disease that may affect Ca^2+^ metabolism (liver and kidney disease, Cushing’s syndrome, bone diseases, malnutrition, and malabsorption conditions) were included. The criterion for healthy individuals was a family history of diabetes.

Laboratory tests

The patients were divided into four groups according to the results of the OGTT and fasting blood glucose (FGT) levels, and individuals with IFG and IGT were considered as prediabetes. Patients with a fasting glucose level of 100-125 mg/dL and an OGTT hour two blood glucose level of 140-199 mg/dL were considered as IGT, those with an FGL of 100-125 mg/dL and an OGTT hour two blood glucose level of <140 mg/dL were considered as IFG, those with an FGL of 100-125 mg/dL and an OGTT hour two blood glucose level of ≥200 mg/dL were considered as type 2 DM, and those with an FGL of <100 mg/dL and an OGTT hour two blood glucose level of <140 mg/dL were considered healthy controls.

During the OGTT, blood was drawn at minute 0 for 25(OH)D and 1,25(OH)_2_D_3_ levels. An oral glucose tolerance test was performed with 75 g glucose in the Central Laboratory OGTT unit at 08:30 after a minimum of eight hours of fasting and a maximum of 14 hours of fasting. An intravenous (IV) cannula was inserted into a forearm surface vein and the patients were allowed to sit in a comfortable chair until the end of the test. Blood samples were drawn through the inserted IV cannula at minutes 0, 30, 60, 90, and 120 for blood glucose measurement, at minutes 0, 60, and 120 for insulin measurement, and at minute 0 for the 25(OH)D level and 1,25(OH)_2_D_3_. The samples obtained for 25(OH)D and 1,25(OH)_2_D_3_ were centrifuged at the end of the test to be run simultaneously, and the plasmas were stored at -80°C until analysis in the endocrinology laboratory of the hospital. The 25(OH)D vitamin was run using the chronotropic method in an Immunocrom brand device. The radio-immune assay method was performed for 1,25(OH)_2_D_3_ using a BioSource brand device, and insulin measurement was performed using the immunoradiometric assay in an Immunotech brand device.

The Homeostasis Model Assessment (HOMA) score was used to assess insulin resistance. The score were calculated as follows: HOMA1-IR = (FPI × FPG)/22.5, and HOMA1-β% = (20 × FPI)/(FPG-3.5).

These formulas indicate insulin resistance (IR) and β-cell function. Fasting plasma insulin (FPI, mUI/L) indicates fasting plasma insulin concentration, and fasting plasma glucose (FPG, mmol/L) indicates fasting plasma glucose concentration.

Statistical analysis

SPSS version 11.5 (SPSS Inc. Chicago, IL, USA) was used for data analysis. Results were presented as mean ± standard deviation and median (minimum-maximum). The normality of distributions was checked. An analysis of variance (ANOVA) test was performed for the difference between groups in normally distributed data, while the Kruskal-Wallis test was used for data that were non-normally distributed. When the p-value obtained in the ANOVA or Kruskal-Wallis test was significant, multiple comparison tests were done to determine which group had the difference. Correlation analysis was performed using Pearson’s or Spearmen’s correlation analysis according to normally or non-normally distributed data. The chi-square test was used for categorical data, and a p-value of <0.05 was considered statistically significant.

## Results

Patients were divided into four groups according to OGTT results and fasting blood glucose levels. Subsequently, these groups were divided into three groups consisting of 25 patients with IFG and IGT, 25 patients with diabetes, and 25 individuals as control. Of these cases, 55 (73.3%) were females and 20 (26.7%) were males.

The mean age was 46.8 ± 8.3 years in the control group, 48.8 ± 7.9 years in the prediabetes group, and 52.8 ± 7.1 years in the diabetes group. The mean HOMA-IR score among the groups included in the study was 1.71 ± 0.81 in the control group, 2.76 ± 1.96 in the prediabetes group, and 3.95 ± 3.43 in the diabetes group. While the diabetes group was significantly higher than the control group, there was no significant difference between the other groups. The mean HOMA-β score in the control group was 113.08 ± 54.49, 87.57 ± 70.75 in the prediabetes group, and 72.69 ± 73.16 in the diabetes group. There was no significant difference between the groups.

The mean level of 25(OH)D among the groups included in the study was 35 ± 16.9 nmol/L in the control group, 44.5 ± 34.5 nmol/L in the prediabetes group, and 35.7 ± 13.1 nmol/L in the diabetes group, without significant difference. The mean level of 1.25(OH)_2_D_3_ was 15.95 ± 8 pg/mL in the control group, 18.4 ± 7.5 pg/mL in the prediabetes group, and 21.5 ± 7.9 pg/mL in the diabetes group. While 25(OH) vitamin D levels were similar between the groups, 1,25(OH)_2_D_3_ vitamin levels were significantly higher in patients with diabetes (Table 1).

**Table 1:** Comparison of 25(OH)D and 1,25(OH)2D3 levels between control, prediabetes, and diabetes groups. P-values of <0.05 are significant.

Independent variable	Group	Group	P-value
25(OH)D	Control	Prediabetes	0.473
		Diabetes	1.000
	Prediabetes	Control	0.473
		Diabetes	0.570
	Diabetes	Control	1.000
		Prediabetes	0.570
1,25(OH)_2_D_3_	Control	Prediabetes	0.784
		Diabetes	0.037
	Prediabetes	Control	0.784
		Diabetes	0.467
	Diabetes	Control	0.037
		Prediabetes	0.467

There was no significant relationship between the HOMA-IR and β scores and vitamin D levels among the groups. When the levels of vitamin 25(OH)D and vitamin 1,25(OH)_2_D_3_ of the diabetes group were compared between the insulin-resistant group and the other group, no significant difference was found. Moreover, no significant difference was found between the HOMA-IR score and 25(OH)D vitamin level in the diabetes group.

When the 25(OH) vitamin D and 1,25(OH)_2_D_3_ vitamin levels and insulin levels at minutes 0, 60, and 120 were compared between the groups, no significant difference was found between vitamin D and insulin levels.

Among all individuals, there was no correlation between the 25(OH) vitamin D and glucose levels at minutes 0, 30, 60, 90, and 120. There was a significant positive relationship between the 1,25(OH)_2_D_3_ vitamin level and glucose levels at minutes 0 (r = 0.340, p = 0.016), 30 (r = 0.328, p = 0.033), 60 (r = 0.298, p = 0.025), and 90 (r = 0.312, p = 0.005). In contrast, there was no correlation between 1,25(OH)_2_D_3_ vitamin and glucose levels at minute 120 (Table 2).

**Table 2:** Correlation between 25(OH)D and 1,25(OH)2D3 levels and glucose levels at 0, 30, 60, 90, and 120 minutes. P-values of <0.05 are significant.

	Glucose at 0 minutes (p-value)	Glucose at 30 minutes (p-value)	Glucose at 60 minutes (p-value)	Glucose at 90 minutes (p-value)	Glucose at 120 minutes (p-value)	N
25(OH)D	0.947	0.999	0.159	0.445	0.950	75
1,25(OH)_2_D_3_	0.016	0.033	0.025	0.005	0.188	75

Furthermore, univariate analysis was performed to investigate the relationships between 25(OH) vitamin D and 1,25(OH)_2_D_3_ vitamin levels and HOMA-IR and HOMA-β scores. The 1,25(OH)_2_D_3_ vitamin levels were positively correlated with HOMA-β in patients with a body mass index (BMI) of ≥30 (r = 0.439; p = 0.049) (Table 3).

**Table 3:** Correlation between 25(OH)D and 1,25(OH)2D3 levels and HOMA scores in patients with a BMI of <30 and ≥30. HOMA-IR = Homeostasis Model Assessment Insulin Resistance; BMI = body mass index

BMI		HOMA-IR (p-value)	HOMA-β (p-value)
<30	25(OH)D	0.539	0.730
	1,25(OH)_2_D_3_	0.787	0.435
≥30	25(OH)D	0.841	0.648
	1,25(OH)_2_D_3_	0.359	0.049

## Discussion

In recent years, studies have shown that other hormones play as much of a role as insulin in the development of type 2 DM [10,11]. Vitamin D receptors (VDRs) have been identified in pancreatic β cells in addition to many cells (macrophage, active lymphocyte, dendritic cells) [12,13]. Calbindin, a vitamin D-dependent calcium-binding protein, is also present in β cells. The expression of calbindin has been shown to protect β cells from cytokine-induced cell death. These VDR-containing tissues are also sites that produce 1,25(OH)_2_D_3_ [14,15]. Vitamin D affects β cells more than alpha cells [16,17] by increasing the insulin response to glucose stimulation. However, it is thought to have no effects on basal insulin secretion [18].

In two studies conducted among non-diabetics, a correlation was found between 25(OH)D concentration and insulin sensitivity [17,19]. Consequently, individuals with vitamin D deficiency have a tendency to develop insulin resistance and diabetes. In this study, the initial vitamin D level did not have any effect on insulin secretion, but there was an improvement in insulin secretion after supplementation [19]. In our study, the basal 25(OH) vitamin D level and HOMA-β score showing insulin sensitivity were compared in control, prediabetic (IFG and IGT), and diabetic groups, but no significant difference was found. Additionally, the absence of an increase in basal 25(OH) vitamin D level and glucose-induced insulin secretion supported that the basal 25(OH) vitamin D level is not a regulator of glucose-induced insulin secretion.

Another study, conducted using the hyperglycemic clamp method in individuals with glucose intolerance, showed a positive correlation between 25(OH)D levels and insulin sensitivity. The study also showed that vitamin D deficiency had negative effects on β cell function [5]. Hence, individuals with vitamin D deficiency had a tendency to develop insulin resistance and diabetes.

A cohort study that included a 17-year follow-up reported an inverse relationship between 25(OH)D levels and type 2 DM incidence, with high vitamin D levels providing protection from type 2 DM [20]. In another study conducted among 5,677 adults between the ages of 40 and 64 years, the 25(OH)D level was shown to be associated with abnormal glucose tolerance and type 2 DM after adjusting for gender, age, obesity, and seasonal changes [21]. In our study, there was no significant difference between 25(OH)D vitamin levels in diabetes, prediabetes, and control groups. However, 1,25(OH)_2_D_3_ vitamin levels were significantly higher in the diabetes group than in the control group.

Evidence of a positive relationship between vitamin D levels and glucose concentrations after oral glucose loading has been shown in studies [22]. In our study, there was no significant difference between the groups in glucose concentrations (minutes 0, 30, 60, 90, 120) after oral glucose loading and 25(OH)D vitamin levels. There was a significant positive relationship between the 1,25(OH)_2_D_3_ vitamin levels and glucose concentrations at minutes 0, 30, 60, and 90.

In another study examining 12,719 prediabetic and nondiabetic adults, a positive correlation was found between low serum 25(OH)D vitamin levels and prediabetes. In our study, no significant relationship was found between 25(OH)D vitamin levels and HOMA-IR, which indicates insulin resistance, in diabetes, prediabetes, and control groups [23].

In a study conducted among 8,958 adult Koreans without diabetes to examine the relationship between HOMA-IR and HOMA-β and 25(OH)D levels, an inverse relationship was found between 25(OH)D levels and HOMA-β, but there was no significant relationship with HOMA-IR [24]. In our study, there was no significant relationship between 25(OH)D vitamin levels and HOMA-IR, which indicates insulin resistance, and HOMA β, which indicates β cell function, in prediabetes and control groups.

In a study that included 980 female and 1,398 male adults without diabetes and in the age range of 35-56 years, a significant relationship was found between high serum 25(OH)D concentrations and a decrease in the risk of type 2 DM in individuals with prediabetes, while this was not the case for those with normal blood sugar levels [25].

In a study conducted in England, serum 25(OH)D concentrations were measured in 350,211 people without diabetes. During a median follow-up of 8.1 years, 6,940 cases of type 2 DM were documented. Higher serum 25(OH)D concentrations were associated with a lower risk of type 2 DM events. Although no relationship was found between vitamin D levels and diabetes in the initial measurements in our study, there is a need for prospective studies supporting that there may be a negative correlation between them in the follow-up of these patients [26].

In a meta-analysis of epidemiological studies by Sobhen et al., it was revealed that the 25(OH)D vitamin level was inversely associated with the risk of type 2 DM and combined type 2 DM and prediabetes in adults. However, this was not significant in the prediabetes period [27]. In a study of 490 adults without prediabetes and type 2 DM at baseline and with complete data at follow-up examinations, the glucose, insulin, and 25(OH)D levels were measured at baseline and after four years. Prediabetes and type 2 DM were defined by results from an OGTT. The study concluded that low 25(OH)D levels may have contributed to the incidence of prediabetes or type 2 DM in Chinese individuals [28].

Increased β-cell apoptosis plays a key role in the pathogenesis of diabetes. The mammalian target of rapamycin (mTOR) signaling pathway plays a critical role in increased β-cell apoptosis. Emerging evidence suggests that vitamin D deficiency is a potential risk factor for diabetes. Hypotheses in recent studies indicate that 1,25(OH)_2_D_3_ can prevent β-cell apoptosis by inhibiting mTOR signaling pathways. Interestingly, 1,25(OH)_2_D_3_ treatment effectively inhibits β-cell apoptosis by increasing the pathological changes in the mTOR signaling pathway induced by high glucose levels and the expression of various apoptosis-regulating inhibitory proteins (DDIT4, TSC1, TSC2), as well as by blocking the mTOR upregulation [29]. In our study, the detection of a significant positive relationship between 1,25(OH)_2_D_3_ vitamin levels and glucose concentrations at minutes 0, 30, 60, and 90 can be interpreted as an effort of the organism to prevent chronic damage of glucose in β cells by responding to the increase in glucose by increasing the levels of 1,25(OH)_2_D_3_, the production of which is tightly controlled by the endocrine system.

1,25(OH)_2_D_3_ provides a potential benefit in the regulation of the mTOR signaling pathway by stimulating DNA-damage-inducible transcript (DDIT4) expression in the treatment of diabetes [29]. The mechanisms of 1,25(OH)_2_D_3_ protecting β cells from apoptosis are still not fully elucidated. Recent studies have determined that mTOR signaling pathways regulate high glucose-induced apoptosis in diabetes [30]. In our study, the fact that the 1,25(OH)_2_D_3_ vitamin level was significantly higher in the diabetes group compared to the control group can be interpreted as an effort of the organism to prevent glucose-induced apoptosis of 1,25(OH)_2_D_3_.

## Conclusions

In our study, no significant difference was found between the groups (control, prediabetes, diabetes) in terms of 25(OH)D levels. Similarly, there was no significant relationship between 25(OH)D levels and insulin sensitivity and resistance between the groups. In fact, when the relationship between insulin resistance and 25(OH)D levels was examined only in individuals with diabetes, no significant difference was found. The fact that the 25(OH)D levels were not associated with diabetes, prediabetes, insulin resistance, insulin sensitivity, insulin secretion, and glucose secretion can lead to the conclusion that vitamin D levels do not have any effects on diabetes or insulin resistance. At the same time, it is also possible to conclude that this result can be related to ethnic origin differences or different DNA sequences (polymorphism) of the *VDR *gene which can create differences in the effects of vitamin D on endocrine. In our study, the fact that there was a significant positive relationship between the 1,25(OH)_2_D_3_ vitamin levels and glucose concentration at minutes 0, 30, 60, and 90 and that the 1,25(OH)_2_D_3_ vitamin levels were higher in the diabetes group than the control group can be interpreted as the organism’s effort to prevent glucose-induced β-cell apoptosis. Moreover, the basal vitamin D levels were measured in patients newly diagnosed with prediabetes and patients with diabetes in our study. In addition, it was shown that there is a need for large-scale prospective studies to investigate the effects of 25(OH)D, particularly 1,25(OH)_2_D_3_ supplementation, on the development of prediabetes and diabetes. All these findings need to be validated by large-scale studies in the Turkish population as well as in different populations.
